# Diffusion-weighted and dynamic contrast-enhanced magnetic resonance imaging after radiation therapy for bone metastases in patients with hepatocellular carcinoma

**DOI:** 10.1038/s41598-021-90065-1

**Published:** 2021-05-17

**Authors:** Ji Hyun Lee, Gyu Sang Yoo, Young Cheol Yoon, Hee Chul Park, Hyun Su Kim

**Affiliations:** 1 Department of Radiology, Samsung Medical Center, Sungkyunkwan University School of Medicine, 81 Irwon-ro, Gangnam-gu, Seoul, 06351 South Korea; 2 Department of Radiation Oncology, Samsung Medical Center, Sungkyunkwan University School of Medicine, 81 Irwon-ro, Gangnam-gu, Seoul, 06351 South Korea

**Keywords:** Radiotherapy, Cancer imaging

## Abstract

The objectives of this study were to assess changes in apparent diffusion coefficient (ADC) and dynamic contrast-enhanced (DCE) magnetic resonance imaging (MRI) parameters after radiation therapy (RT) for bone metastases from hepatocellular carcinoma (HCC) and to evaluate their prognostic value. This prospective study was approved by the Institutional Review Board. Fourteen patients with HCC underwent RT (30 Gy in 10 fractions once daily) for bone metastases. The ADC and DCE-MRI parameters and the volume of the target lesions were measured before (baseline) and one month after RT (post-RT). The Wilcoxon signed-rank test was used to compare the parameters between the baseline and post-RT MRI. The parameters were compared between patients with or without disease progression in RT fields using the Mann–Whitney test. Intraclass correlation coefficients were used to evaluate the interobserver agreement. The medians of the ADC, rate constant [*k*_ep_], and volume fraction of the extravascular extracellular matrix [*v*_*e*_] in the baseline and post-RT MRI were 0.67 (range 0.61–0.72) and 0.75 (range 0.63–1.43) (× 10^–3^ mm^2^/s) (*P* = 0.027), 836.33 (range 301.41–1082.32) and 335.80 (range 21.86–741.87) (× 10^–3^/min) (*P* = 0.002), and 161.54 (range 128.38–410.13) and 273.99 (range 181.39–1216.95) (× 10^–3^) (*P* = 0.027), respectively. The medians of the percent change in the ADC of post-RT MRI in patients with progressive disease and patients without progressive disease were − 1.35 (range − 6.16 to 6.79) and + 46.71 (range 7.71–112.81) (%) (*P* = 0.011), respectively. The interobserver agreements for all MRI parameters were excellent (intraclass correlation coefficients > 0.8). In conclusion, the ADC, *k*_ep_, and *v*_*e*_ of bone metastases changed significantly after RT. The percentage change in the ADC was closely related to local tumor progression.

## Introduction

Hepatocellular carcinoma (HCC) is the sixth most common cancer diagnosed worldwide and is the third leading cause of cancer-related mortality^1^. Despite advances in diagnostic imaging modalities and treatment strategies, some patients present with tumor progression in the form of skeletal metastasis^2^, most commonly in the spine^3^. This may result in skeletal-related events, including pathologic fracture, spinal cord compression, and neurologic deficits, causing significant deterioration in patients’ quality of life^2^. Therefore, in addition to the control of the primary tumor, appropriate management of bone metastases is considered mandatory in patients with HCC.

Although the standard treatment for metastatic HCC is systemic therapy with drugs such as sorafenib and lenvatinib, the tumor responses are not satisfactory^4–6^. In HCC patients with bone metastases, radiation therapy (RT) is widely used for local palliation or the prevention of disease aggravation^7^. However, patients experience diverse tumor responses, and there is a significant rate of retreatment after conventional RT, which can be attributed to local tumor progression^8–10^. Hence, a radiological tool predicting tumor progression after RT for bone metastasis in HCC patients can aid in the selection of patients who may require supplementary therapy after conventional RT.

Among various advanced MRI techniques, diffusion-weighted (DW) and dynamic contrast-enhanced (DCE) MRI, which reflect the diffusion and perfusion properties of the tissues, respectively, have shown potential in providing noninvasive quantitative information about tumor cellularity, biological aggressiveness, microenvironment, and angiogenesis^11–13^. In musculoskeletal imaging, DW imaging has been reported to be useful for tumor characterization^14–16^ and treatment response evaluation^17–20^. DCE-MRI evaluates tumor perfusion and vascularity^21,22^. In particular, quantitative estimations of the parameters using pharmacokinetic models have shown promising results in terms of their correlation with the histologic features of tumors and the clinical parameters in the musculoskeletal region^23,24^.

Radiation causes endothelial damage or alters angiogenic pathways, leading to the disruption of the vascular structure and changing the blood flow of tumors^25,26^. Therefore, we hypothesized that RT alters the hemodynamics and vascular characteristics as well as the tumor cellularity of bone metastases from HCC, which may be detected using DW- and DCE-MRI. In this context, this study aimed to evaluate changes in DW- and DCE-MRI parameters of bone metastases in patients with HCC after RT. Their prognostic value in predicting local tumor progression was also assessed.

## Materials and methods

This prospective study was approved by the Institutional Review Board (Samsung Medical Center, IRB File No. 2018-07-159), registered at cris.nih.go.kr (KCT0004861), and was conducted from February 2019 to July 2020. Written informed consent was obtained from all patients and the study was conducted in accordance with the declaration of Helsinki.

### Patients

According to a previous study^27^, the volume transfer constant (*K*^trans^) after RT was expected to decrease by 33.5% (standard deviation 28.9%). Using paired t-tests, the required sample size was calculated to be 10 in order to have a 90% chance of finding an average of 33.5% difference at a significance level of 5% (α = 0.05, β = 0.10) (MedCalc Statistical Software version 19.1.5; MedCalc Software Ltd.). Therefore, assuming a dropout rate of 25%, a total of 14 patients were required.

Patients with HCC who were scheduled to undergo RT for bone metastases were included if they (a) were older than 18 years, (b) had a histopathological or imaging diagnosis of HCC, and (c) had metastases in the thoracic or lumbosacral spine or pelvic bone; patients with metastatic lesions in other locations were not included to minimize unwanted contributions from any potential region-dependent biases. The criteria for considering bone metastases from HCC included newly developed or progressed bone lesions noted during computed tomography (CT) surveillance or histopathological confirmations by bone biopsies, where available. The exclusion criteria were as follows: (a) another primary malignancy, (b) history of surgery, RT, or metallic instrumentation at the above metastatic sites; (c) contraindication to gadolinium-based contrast agents or MRI examinations, or (d) pregnant or nursing female patients. We collected clinical information, including sex, age, etiology of HCC, Child–Pugh score, serum α-fetoprotein level, status of liver cirrhosis, concurrent systemic therapy, and survival.

### Radiation therapy

All patients underwent RT with 30 Gy in ten fractions with once daily schedule. The simulation with contrast-enhanced CT using a 2.5–5-mm slice thickness was performed within three days before starting RT, on the same day as baseline MRI, following which RT was initiated. The gross tumor volume was delineated according to baseline MRI. The clinical target volume included the gross tumor volume and the adjacent segments of the spine. The planning target volume was defined as an isotropic extension of 5 mm from the clinical target volume. Both X-ray and proton beam therapies were used in this study. The registration of the simulation CT, target volume delineation, and calculation of dosimetry were performed using Pinnacle version 9.10 (Philips Radiation Oncology Systems) for X-ray RT and RayStation (RaySearch Laboratories) for proton beam therapy. An orthogonal X-ray image was obtained in the treatment room for image verification before RT.

### MRI examinations

All examinations were performed using a 3.0-T MRI scanner (Ingenia; Philips Medical Systems). MRI was performed before (baseline) and one month after completing RT (first post-RT; range 15–45 days). Conventional MRI sequences consisted of turbo spin-echo axial T1-weighted imaging (T1WI), T2-weighted image (T2WI), sagittal T2WI, and sagittal (spine) or coronal (pelvic bone) T1WI. Axial plane DW-MRI was obtained using a single-shot echoplanar sequence as follows: fat suppression method, chemical shift selective saturation; phase encoding direction, anteroposterior; number of averages, 3; parallel imaging, SENSE with a reduction factor of 2; water-fat shift, 10.84 pixels; interpolated voxel size, 1.367 mm; slice gap, 0 mm; breath-hold or triggering, none; diffusion time (Δ), 26.5 ms; length of the gradient pulse (δ), 16.06 ms. Sensitizing diffusion gradients were applied sequentially in the x, y, and z directions using *b* values of 0, 400, and 1400^28,29^. ADC maps were generated automatically on the main MRI console with a mono-exponential fitting of the three selected *b* values. DCE-MRI was performed using a three-dimensional fast field-echo sequence in the axial plane. Before injecting the contrast material, the pre-contrast T1-weighted fast field-echo sequences (flip angles, 5° and 10°) were applied according to the same geometry to calculate the baseline T1 maps with the same axial three-dimensional fast field-echo sequence. DCE-MRI was performed immediately after injecting a bolus of gadoterate meglumine (Dotarem^®^; Guerbet) at a rate of 3 mL/s and a dose of 0.1 mmol/kg, followed by a 15-mL flush of normal saline. DCE-MRI included 1050 dynamic images with a temporal resolution of 4.3 s obtained over 5 min (flip angle, 15°; parallel imaging, SENSE with a reduction factor of 2; breath-hold or triggering, none; fat suppression, none). Contrast-enhanced T1WIs were collected after DCE imaging in the axial and sagittal planes of the thoracic or lumbosacral spine and in the axial and coronal planes of the pelvic bone (Table 1). Three months after completing RT, follow-up MRI was performed using conventional sequences (second post-RT; range 70–100 days).

**Table 1:** Parameters of the magnetic resonance sequences.

	Axial T1WI	Axial T2WI	Sagittal T2WI	Sagittal T1WI^a^	Coronal T1WI^b^	Axial DWI	Axial DCE image	Postcontrast sagittal T1WI^a^	Postcontrast coronal T1WI^b^	Postcontrast axial T1WI
TR (ms)	480.1–736.6	7042–16,361	3000–3159	609–830	540.8	2677.7–5710.4	13	468.2	451	609
TE (ms)	7.4–10	120	80–128	10	15	78	1.76	15	15	10
Acquisition matrix	380 × 268–452 × 446	379 × 223–452 × 446	500 × 251–951 × 472	500 × 251–951 × 472	780 × 384	140 × 196	128 × 239	500 × 251–951 × 472	780 × 384	380 × 268–452 × 446
FOV (cm)	36–38	36–38	28–36	28–36	35	35	35	28–36	35	36–38
Thickness (mm)	2.5	2.5	4	4	5	5–8	10	4	5	2.5
Acquisition time	5 min 12 s	3 min 49 s	1 min 53 s–4 min 30 s	3 min 32 s–5 min 16 s	2 min 45 s	1 min 49 s–3 min 55 s	5 min 34 s	3 min 9 s–5 min 58 s	2 min 15 s	5 min 11 s
*b* values						0, 400, 1400				

*TR* repetition time, *TE* echo time, *FOV* field of view, *T1WI* T1-weighted image, *T2WI* T2-weighted image, *DWI* diffusion-weighted image, *DCE* dynamic contrast-enhanced.

^a^Thoracic or lumbosacral spine.

^b^Pelvic bone.

### Image analysis

DCE-MRI maps were generated using image-processing software (IntelliSpace Portal version 10.0; Philips). The signal intensity on MRI was converted into an equivalent concentration of contrast material using the variable flip angle method^30^. DCE parameters (*K*^trans^, rate constant [*k*_ep_], volume fraction of the extravascular extracellular matrix [*v*_*e*_], and blood plasma volume [*v*_*p*_]) were estimated using the extended Tofts model^31^ with the population-averaged arterial input function (AIF)^32^.

**Figure 1:**
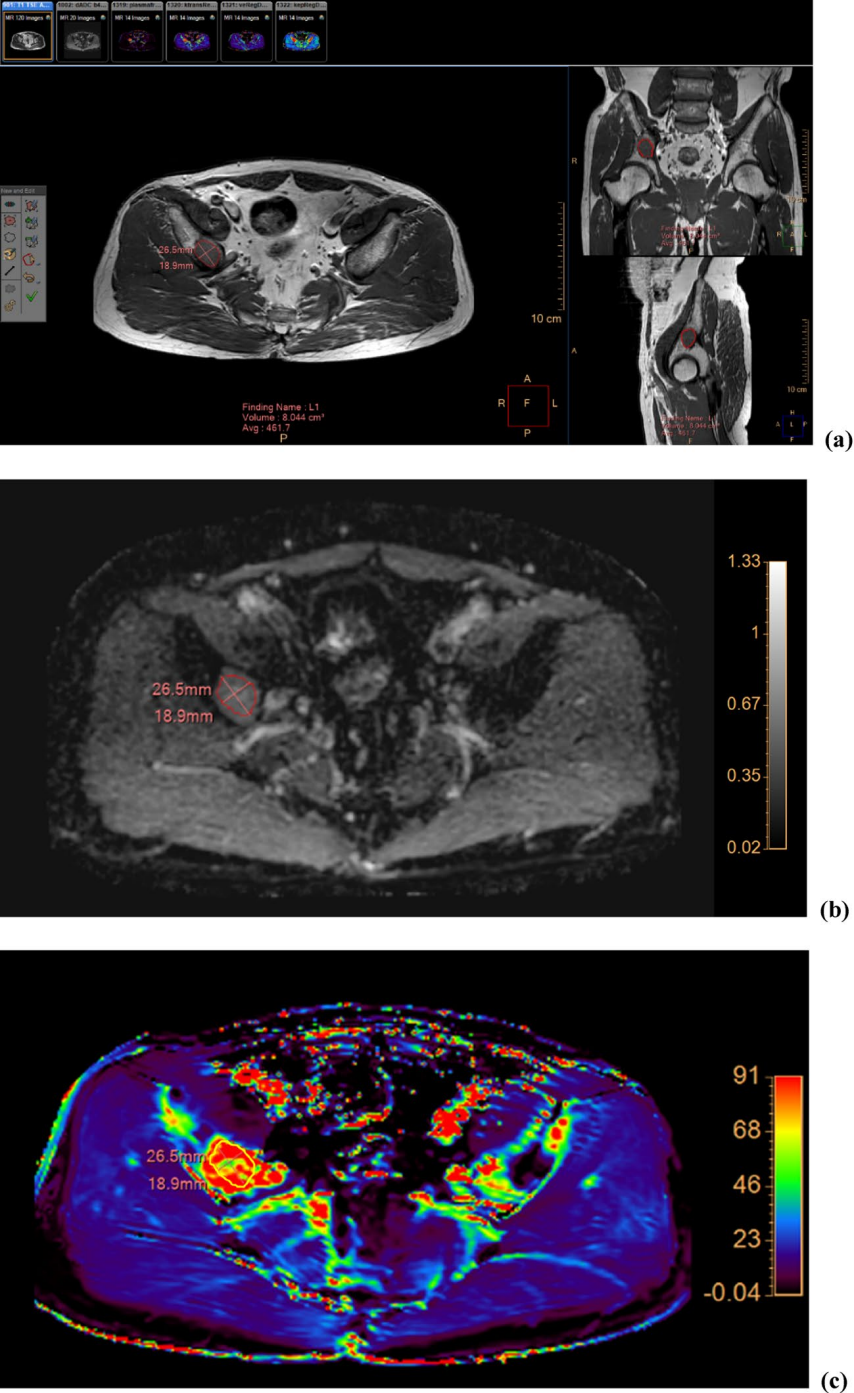

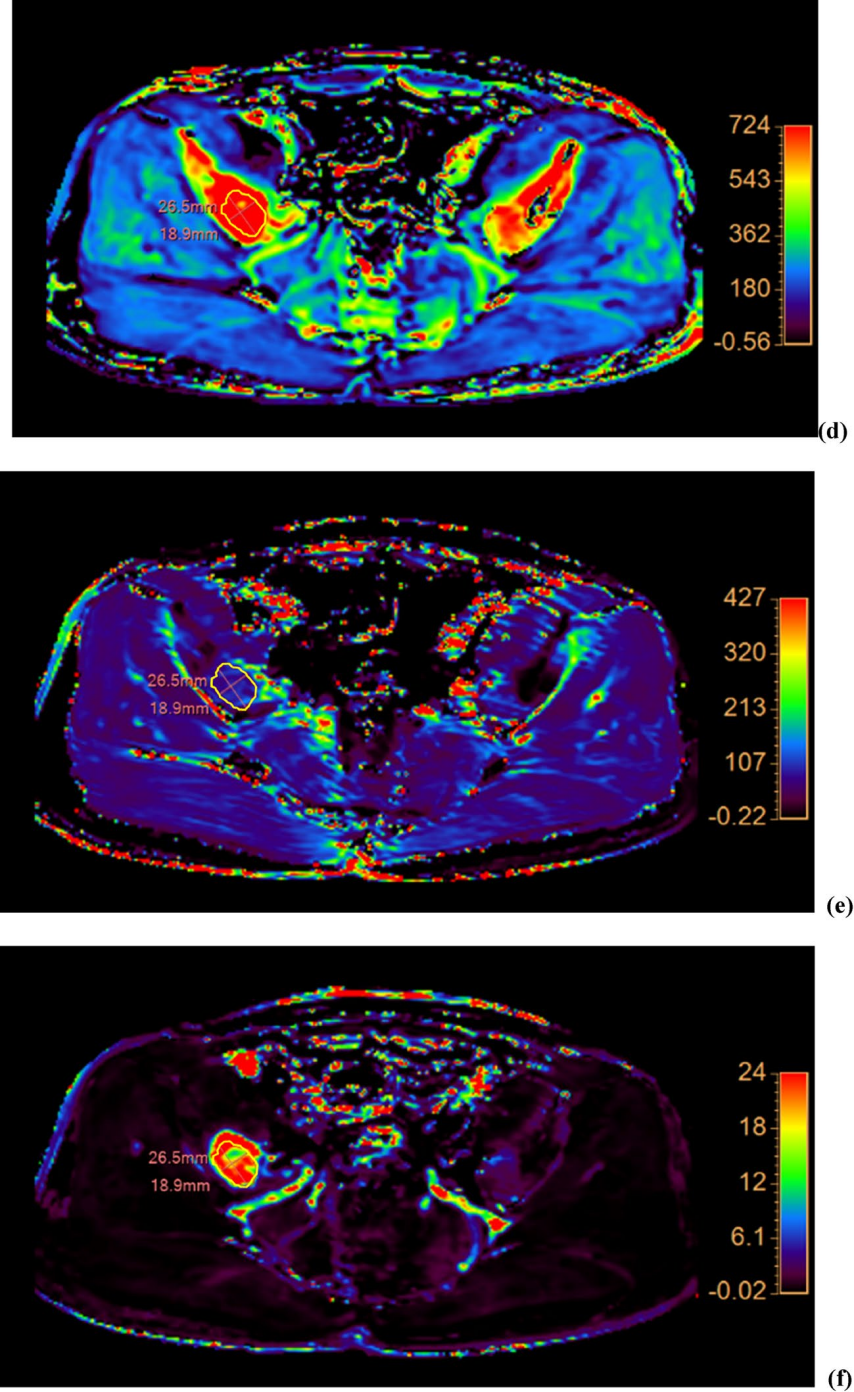
Images of a 64-year-old man with hepatocellular carcinoma showing metastasis to the right iliac bone. (**a**) The ROI was drawn within the lesion encompassing the whole tumor volume on axial T1-weighted images and reformatted coronal (right upper panel) and sagittal (right lower panel) images. The corresponding ADC map (**b**) and color-encoded overlay maps of the *K*^trans^ (**c**), *k*_ep_ (**d**), *v*_*e*_ (**e**), and *v*_*p*_ (**f**) are shown with ROIs and scale bars. ADC, apparent diffusion coefficient; *K*^trans^, volume transfer constant; *k*_ep_, rate constant; *v*_*e*_, volume fraction of the extravascular extracellular matrix; *v*_*p*_, blood plasma volume; ROI, region of interest.

Two independent radiologists (readers I and II with 15 and 5 years of experience in musculoskeletal MRI, respectively) who were blinded to clinical information performed the tumor segmentation on anatomic reference images; the axial T1WI, T2WI, and postcontrast T1WI, in which tumor margins were most clearly delineated, were selected. The reference image, ADC, and DCE parameter maps were loaded into a multimodality tumor tracking application (IntelliSpace Portal version 10.0; Philips). After selecting the regions of interest (ROI) using the “smart ROI” tool with edge detection, the ROIs were automatically propagated in the craniocaudal direction (Fig. 1). Manual adjustments were performed to ensure accuracy in encompassing the whole tumor volume, including both intraosseous and extraosseous components. Adjacent vertebral endplates or intervertebral discs were carefully avoided. ROIs drawn on anatomic reference images were simultaneously and automatically drawn on the corresponding location on ADC and DCE parameter maps. The mean values of ADC, *K*^trans^, *k*_ep_, *v*_*e*_, *v*_*p*_, and volume from the volumetric ROI were recorded.

### Evaluation of treatment response

The local tumor response was evaluated according to the MD Anderson criteria (complete response [CR], partial response [PR], progressive disease [PD], and stable disease [SD])^33^. The patients were categorized into CR, PR, PD, or SD as follows: CR, normalization of signal intensity; PR, ≥ 50% decrease in size; PD, ≥ 25% increase in size; and SD, < 25% increase or < 50% decrease in size. The measurements were based on the sum of the perpendicular bi-dimensional measurements of the greatest diameters of each lesion in the baseline and second post-RT MRIs analyzed by reader II. Patients with CR, PR, and SD were regarded as the non-PD group, and those with PD were regarded as the PD group.

Pain status was assessed using the numeric rating scale (NRS) score three days before initiating RT, during the course of RT, and one and three months after completing RT. To evaluate the pain response after RT, the categories of the International Bone Metastases Consensus Group were used to adjust the confounding effects of analgesics^34^. To apply these categories, we calculated the oral morphine equivalent dose (OMED) of all analgesics administered to patients before and after RT. Neurological symptoms were graded according to the neurological grading system for spinal cord compression by metastatic tumor^35^. Toxicities related to the treatment were evaluated according to the Common Terminology Criteria for Adverse Events version 5.0.

### Statistical analysis

The Wilcoxon signed-rank test was used to determine whether MRI parameters in the first post-RT MRI were different from those in the baseline MRI. Changes in ADC, *K*^trans^, *k*_ep_, *v*_*e*_, *v*_*p*_, and volume in the first post-RT MRI, defined as the percentage change from baseline values, were calculated (ΔADC%, Δ*K*^trans^%, Δ*k*_ep_%, Δ*v*_*e*_%, Δ*v*_*p*_%, and Δvolume%, respectively). Patient characteristics were compared between the non-PD and PD groups; the continuous and categorical variables were analyzed using the Mann–Whitney test and Fisher’s exact test, respectively. For continuous variables with *P* values < 0.20, a receiver operating characteristic (ROC) curve was constructed, and the area under the curve (AUC) was calculated. The optimal cutoff points were based on the maximum Youden index.

The interobserver agreement between readers I and II was assessed using the intraclass correlation coefficient (ICC). An ICC of 1.0 was considered to represent perfect agreement; 0.81–0.99, almost perfect agreement; 0.61–0.80, substantial agreement; 0.41–0.60, moderate agreement; 0.21–0.40, fair agreement; and 0.20 or less, slight agreement^36^.

All statistical analyses were performed using MedCalc Statistical Software version 19.4.0, and *P* values < 0.05 were considered statistically significant.

## Results

Among 14 patients, four were excluded for the following reasons: withdrawal of consent (*n* = 2), inability to undergo MRI examination owing to a deterioration in his/her general condition (*n* = 1), and inappropriate MRI acquisition (*n* = 1). Ten patients were finally included. Proton beam therapy was performed in only one patient among the ten patients. The median age and follow-up duration were 63 years (range 43–73 years) and 6 months (range 3–7 months), respectively. The median time interval between completing RT and the first post-RT MRI was 30 days (range 23–34 days). Four patients experienced PD of the target lesions in the second post-RT MRI and two died of disease progression. There was no significant difference in clinical variables between the PD and non-PD groups (Table 2).

**Table 2:** Comparison of clinical variables between the non-PD and PD groups.

	Non-PD group (*%*)	PD group (%)	*P* value
**Sex**			1.000
Male	5 (83.3)	4 (100.0)	
Female	1 (16.7)	0 (0.0)	
**Etiology of HCC**			0.679
Hepatitis B	4 (66.7)	3 (75.0)	
Hepatitis C	1 (16.7)	1 (25.0)	
Others	1 (16.7)	0 (0.0)	
**Accompanying liver cirrhosis**			1.000
Yes	3 (50.0)	2 (50.0)	
No	3 (50.0)	2 (50.0)	
**Child–Pugh class**			0.400
A5	6 (100.0)	3 (75.0)	
A6	0 (0.0)	1 (25.0)	
**Location of target lesion**			0.172
Thoracic spine	3 (50.0)	0 (0.0)	
Lumbar spine	3 (50.0)	2 (50.0)	
Sacrum	0 (0.0)	1 (25.0)	
Ilium	0 (0.0)	1 (25.0)	
**Radiation therapy modality**			0.400
X-ray therapy	6 (0.0)	3 (75.0)	
Proton therapy	0 (0.0)	1 (25.0)	
**Concurrent systemic treatment**			0.400
Yes	0 (0.0)	1 (25.0)	
No	6 (100.0)	3 (75.0)	
**Median αFP (ng/mL)**^a^	10 (5.3–41,306.2)	4056.6 (3.4–156,059)	1.000

*PD* progressive disease, *HCC* hepatocellular carcinoma, *αFP* alpha-fetoprotein.

^a^Numbers in parentheses are ranges.

**Figure 2:**
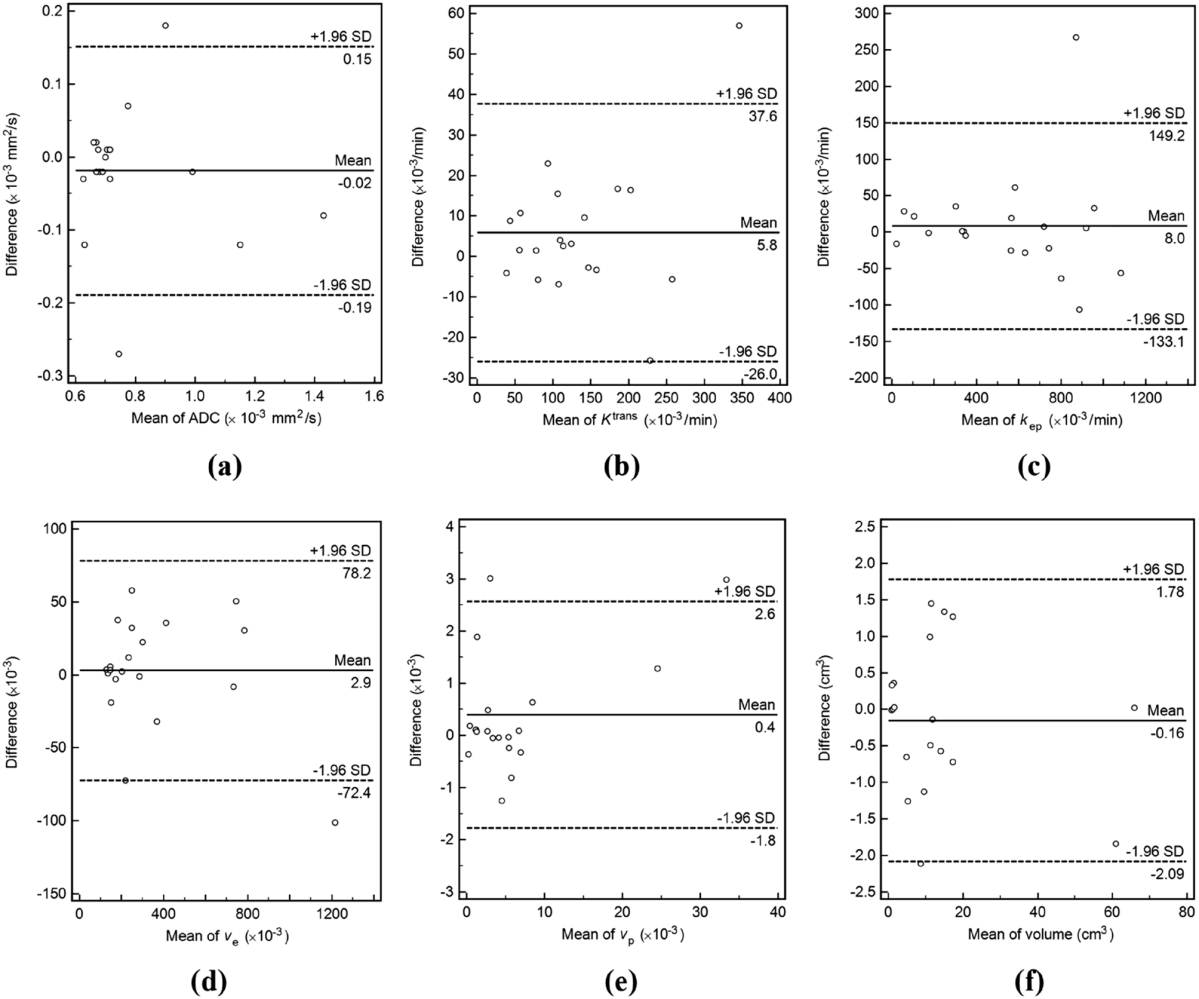
Bland–Altman plots of (**a**) ADC, (**b**) *K*^trans^, (**c**) *k*_ep_, (**d**) *v*_*e*_, (**e**) *v*_*p*_, and (**f**) tumor volume demonstrating agreement between the values measured by the two readers. The difference (*y*-axis) between the measurements obtained by the two readers is plotted against the mean value (*x*-axis) of the measurements obtained by them. The solid line and the top and bottom dashed lines indicate the mean difference and the upper and lower margins of 95% limits of agreement, respectively. ADC, apparent diffusion coefficient; *K*^trans^, volume transfer constant; *k*_ep_, rate constant; *v*_*e*_, volume fraction of the extravascular extracellular matrix; *v*_*p*_, blood plasma volume; SD, standard deviation.

The interobserver agreements were as follows: ADC ICC = 0.912, 95% confidence interval [CI] 0.794–0.964; *K*^trans^ ICC = 0.977, 95% CI 0.943–0.991; *k*_ep_ ICC = 0.976, 95% CI 0.942–0.991; *v*_*e*_ ICC = 0.992, 95% CI 0.979–0.997; *v*_*p*_ ICC = 0.999, 95% CI 0.996–0.999; volume ICC = 0.999, 95% CI 0.996–0.999. Because the measurements of all MRI parameters showed almost perfect interobserver agreement, the average of both readers’ measurements was used (Fig. 2).

**Figure 3:**
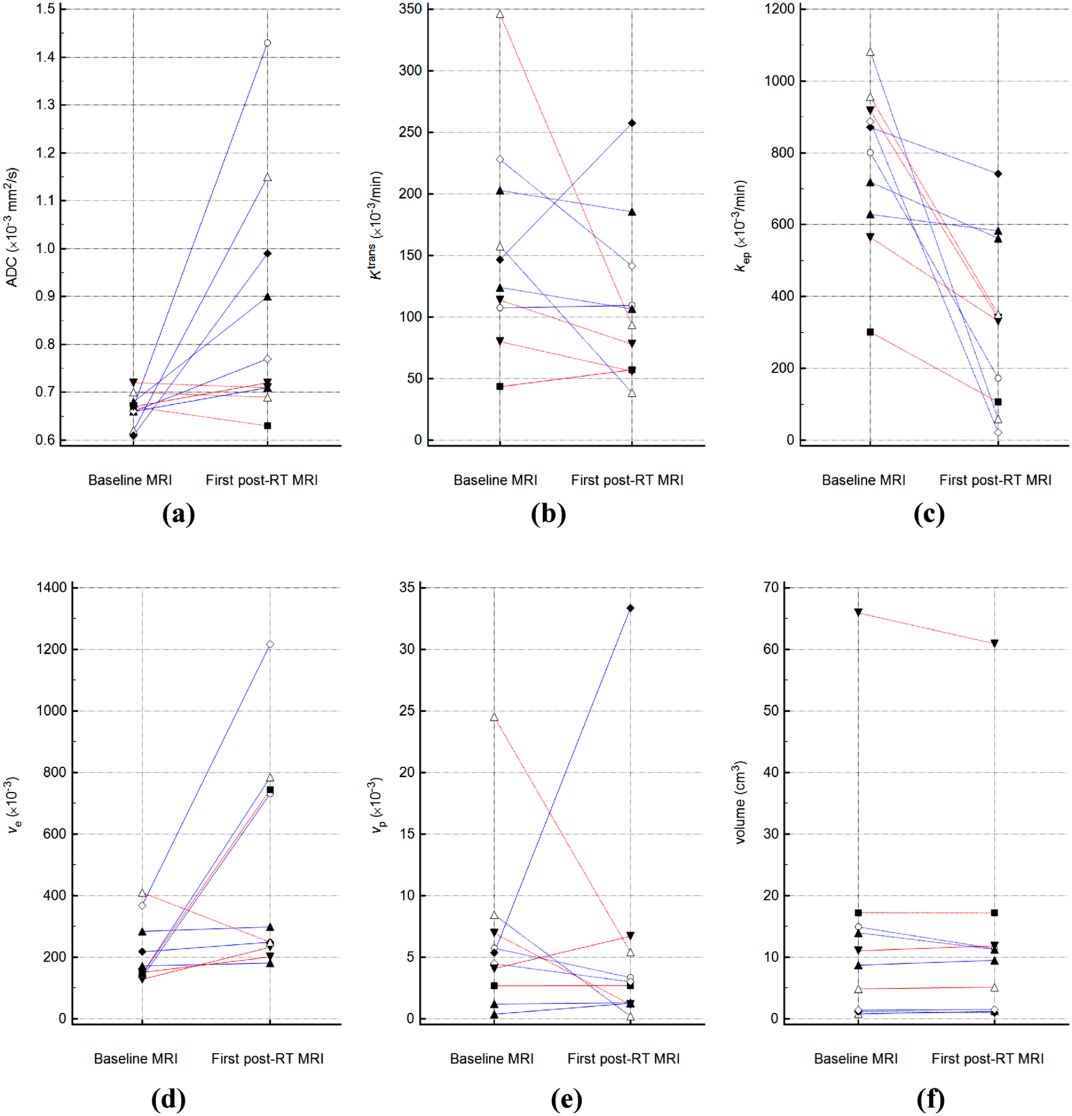
Graphs showing changes in (**a**) ADC, (**b**) *K*^trans^, (**c**) *k*_ep_, (**d**) *v*_*e*_, (**e**) *v*_*p*_, and (**f**) tumor volume following RT for each patient. The increasing values are represented by solid lines and the decreasing ones by dashed lines. The data of the non-PD and PD groups are represented by blue and red lines, respectively. ADC, apparent diffusion coefficient; *K*^trans^, volume transfer constant; *k*_ep_, rate constant; PD, progressive disease; RT, radiation therapy; *v*_*e*_, volume fraction of the extravascular extracellular matrix; and *v*_*p*_, blood plasma volume.

**Figure 4:**
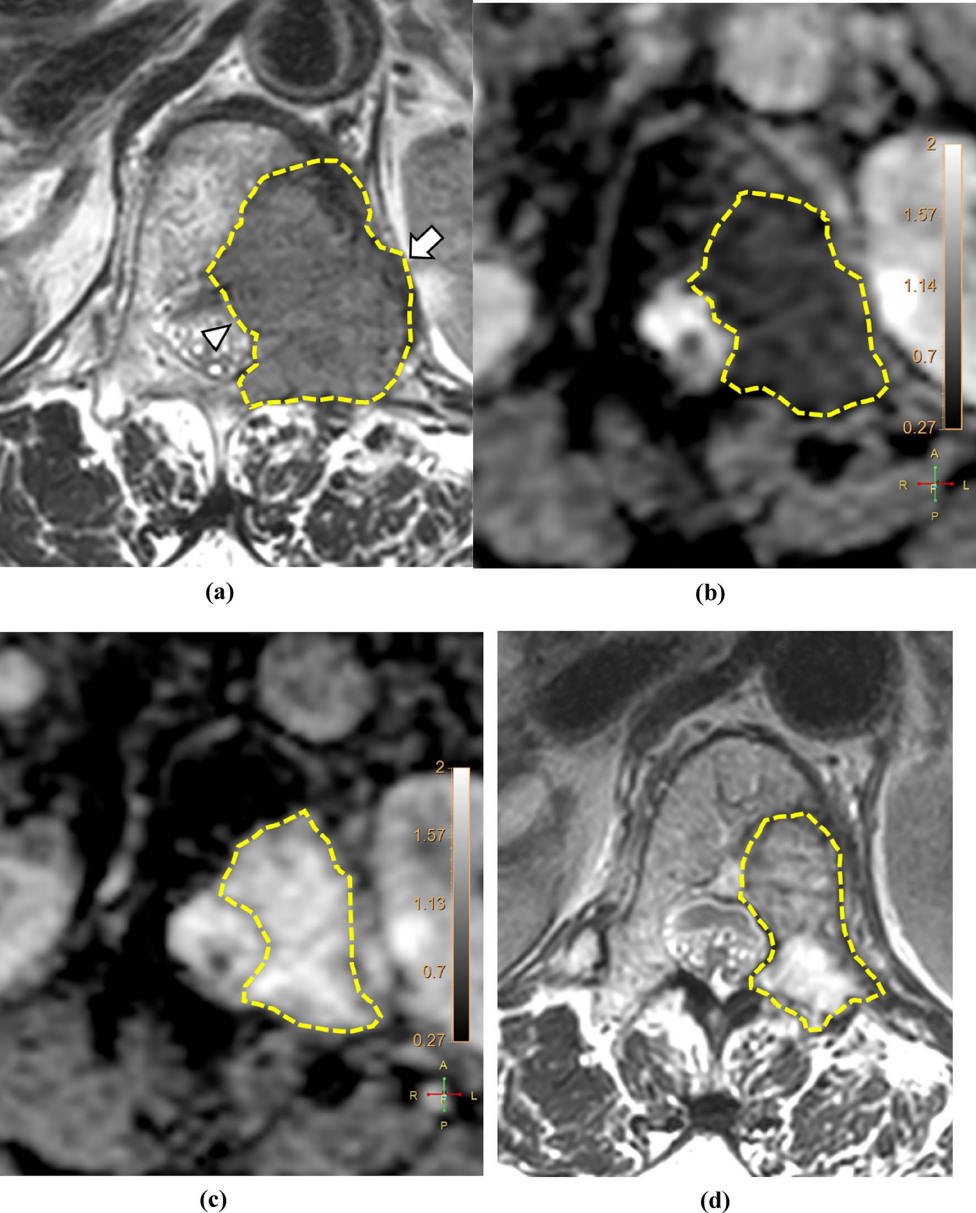
Images of a 71-year-old woman with metastasis to the T12 vertebra from a hepatocellular carcinoma. The tumor margin is delineated by yellow dotted lines. (**a**) The T2WI of the baseline MRI shows a 2.6 × 3.7-cm-sized metastatic lesion with an extraosseous extension to the left paravertebral (arrow) and epidural spaces (arrowhead). Compared with (**b**) the baseline MRI, the average mean ADC value measured by readers I and II in the (**c**) first post-RT MRI increased from 0.68 to 1.39 (× 10^–3^ mm^2^/s). (**d**) The T2WI of the second post-RT MRI shows that the size of the lesion decreased to 1.6 × 3.4 cm, representing a partial response. *ADC* apparent diffusion coefficient, *MRI* magnetic resonance imaging, *RT* radiation therapy, *T2WI* T2-weighted image.

**Figure 5:**
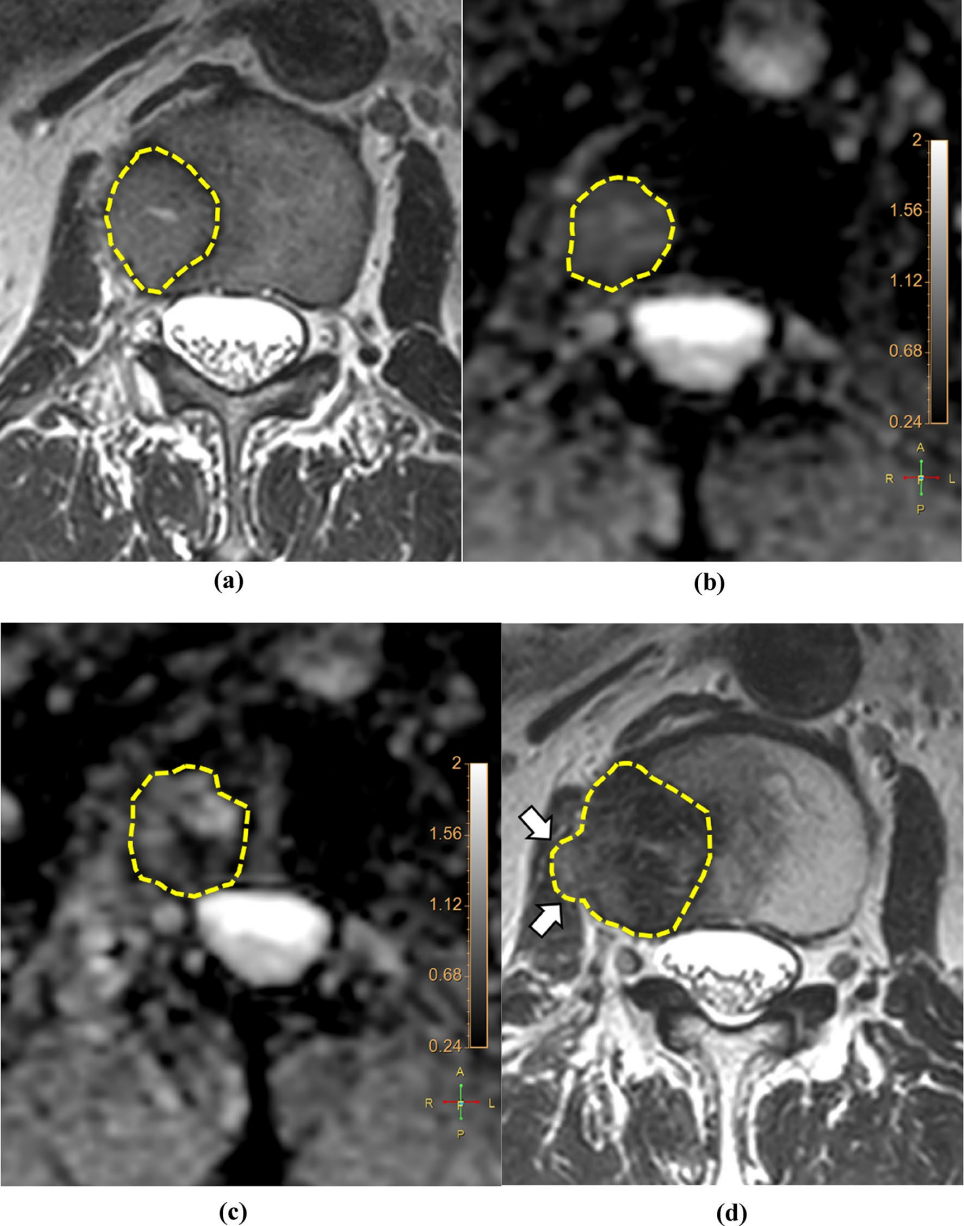
Images of a 72-year-old man with metastasis to the L2 vertebra from a hepatocellular carcinoma. The tumor margin is delineated by yellow dotted lines. (**a**) The T2WI of the baseline MRI shows a 1.8 × 2.3-cm-sized metastatic lesion. The average mean ADC values measured by readers I and II in (**b**) the baseline MRI and (**c**) the first post-RT MRI were 0.72 and 0.71 (× 10^–3^ mm^2^/s), respectively. (**d**) The T2WI of the second post-RT MRI shows an increase in the size of the lesion to 2.6 × 2.7 cm with extraosseous extension (arrows), representing PD. *ADC* apparent diffusion coefficient, *MRI* magnetic resonance imaging, *RT* radiation therapy, *T2WI* T2-weighted image, *PD* progressive disease.

ADC, *k*_ep_, and *v*_*e*_ in the first post-RT MRI were significantly different from those in the baseline MRI, with changes of + 31.65% ± 41.52%, -54.70 ± 32.21%, and + 161.93 ± 198.47% [mean ± standard deviation], respectively (Table 3, Figs. 3, 4, 5). While *K*^trans^, *v*_*p*_, and volume changed by − 16.16 ± 45.60%, + 49.74 ± 191.81%, and + 2.03 ± 21.98%, respectively, these changes were not statistically significant.

**Table 3:** MRI parameters before and after RT.

	Baseline MRI	First post-RT MRI	*P* value
ADC (× 10^–3^ mm^2^/s)	0.67 (0.61–0.72)	0.75 (0.63–1.43)	0.027
*K*^trans^ (× 10^–3^/min)	135.38 (43.51–346.04)	100.08 (38.62–257.49)	0.106
*k*_ep_ (× 10^–3^/min)	836.33 (301.41–1082.32)	335.80 (21.86–741.87)	0.002
*v*_*e*_ (× 10^–3^)	161.54 (128.38–410.13)	273.99 (181.39–1216.95)	0.027
*v*_*p*_ (× 10^–3^)	4.93 (0.38–24.52)	2.86 (0.21–33.35)	0.625
Volume (cm^3^)	9.90 (0.80–65.93)	10.40 (1.01–60.93)	0.770

Numbers are medians and ranges in parentheses.

MRI, magnetic resonance imaging; ADC, apparent diffusion coefficient; *K*^trans^, volume transfer constant;* k*_ep_, rate constant; RT, radiation therapy; *v*_*e*_, volume fraction of the extravascular extracellular matrix; *v*_*p*_, blood plasma volume.

**Figure 6:**
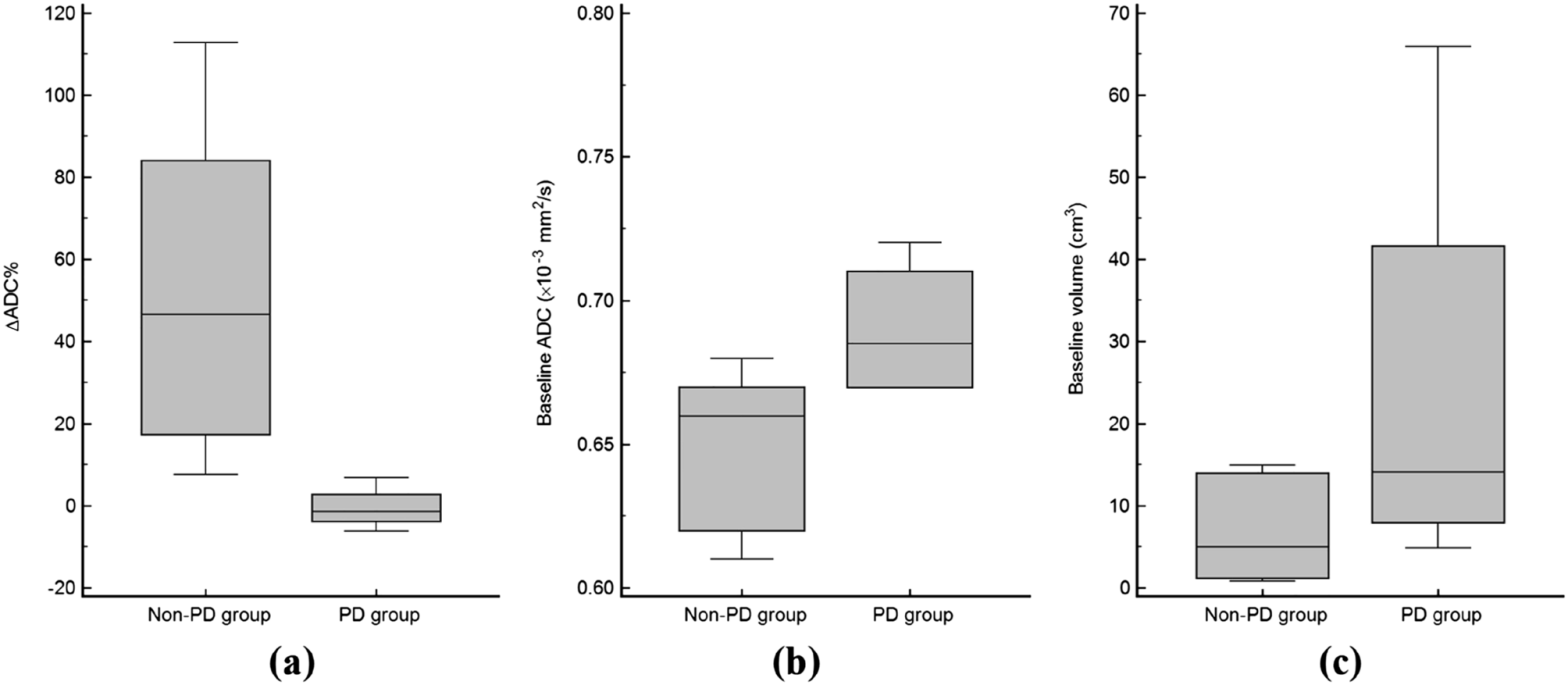
Boxplots for (**a**) ΔADC%, (**b**) baseline ADC, and (**c**) baseline volume in the non-PD and PD groups. The top and bottom of the box denote the 25th and 75th percentiles, respectively. The mid lines and bars indicate the medians and 5th–95th percentiles, respectively. *ADC* apparent diffusion coefficient, *PD* progressive disease.

The PD group showed a significantly lower ΔADC% than the non-PD group (Table 4, Fig. 6). The baseline ADC and baseline volumes were not significantly different between the two groups. The AUCs of ΔADC%, baseline ADC, and baseline volume for differentiating between the non-PD and PD groups were 1.000 (95% CI 0.692–1.000), 0.875 (95% CI 0.525–0.994), and 0.792 (95% CI 0.435–0.972), respectively. The cutoffs for ΔADC%, baseline ADC, and baseline volume were 6.79% (sensitivity, 100.0%; specificity, 100.0%), 0.66 × 10^–3^ mm^2^/s (sensitivity, 100.0%; specificity, 66.7%), and 1.39 cm^3^ (sensitivity, 100.0%; specificity, 50.0%), respectively.

**Table 4:** Comparison of MRI parameters between the non-PD and PD groups.

	Non-PD group (n = 6)	PD group (n = 4)	*P* value
ADC (× 10^−3^ mm^2^/s)^a^	0.66 (0.61–0.68)	0.69 (0.67–0.72)	0.051
*K*^trans^ (× 10^−3^/min)^a^	152.20 (107.47–228.01)	96.94 (43.51–346.04)	0.286
*k*_ep_ (× 10^−3^/min)^a^	836.33 (628.82–1082.32)	741.29 (301.41–956.28)	0.670
*v*_*e*_ (× 10^−3^)^a^	195.00 (135.30–368.89)	148.30 (128.38–410.13)	0.522
*v*_*p*_ (× 10^−3^)^a^	4.93 (0.38–8.46)	5.53 (2.38–24.52)	0.522
Volume (cm^3^)^a^	5.06 (0.80–14.93)	14.16 (4.88–65.93)	0.136
ΔADC%	46.71 (7.71–112.81)	− 1.35 (− 6.16 to 6.79)	0.011
Δ*K*^trans^%	− 11.31 (− 75.51 to 75.54)	− 30.74 (− 72.97 to 31.48)	0.670
Δ*k*_ep_%	− 50.11 (− 97.54 to − 7.29)	− 63.20 (− 64.85 to − 41.27)	1.000
Δ*v*_*e*_%	122.14 (5.17–440.48)	57.46 (− 39.27 to 410.41)	0.670
Δ*v*_*p*_%	− 11.51 (− 97.55 to 520.64)	− 38.44 (− 83.39 to 64.20)	0.522
ΔVolume%	-3.26 (− 23.63 to 53.20)	2.61 (− 7.58 to 7.36)	1.000

Numbers are medians and ranges in parentheses.

MRI, magnetic resonance imaging; ADC, apparent diffusion coefficient, *K*^trans^, volume transfer constant; *k*_ep_, rate constant; PD, progressive disease; *v*_*e*_, volume fraction of the extravascular extracellular matrix; *v*_*p*_, blood plasma volume.

^a^Data from baseline MRI.

Only three (30%) patients complained of pain, with NRS scores of four (*n* = 1) and three (*n* = 2), while the other seven patients did not have any pain relevant to the target lesions and were not administered any analgesics. The median OMED of the three patients with relevant pain was 24 mg (range 7.5–30 mg). After completing RT, two patients had complete remission of pain and one had partial remission. Among the seven patients without any relevant pain before RT, two developed post-RT pain that was related to the PD of the target lesion. Only one patient had neurological symptoms graded as b, showing radiculopathy, which was relevant to the target lesion. The neurological symptoms were relieved one month after RT; however, relapse was observed owing to PD of the target lesion. No grade 3 or 4 toxicities were observed during the follow-up (Table 5).

**Table 5:** Toxicity profiles related to radiation therapy.

Toxicities	Grade 1 (%)	Grade 2 (%)	Total (%)
Anorexia	1 (10.0)	1 (10.0)	2 (20.0)
Nausea	1 (10.0)	0 (0.0)	1 (10.0)
Diarrhea	2 (0.0)	0 (0.0)	2 (20.0)

## Discussion

We evaluated changes in DW- and DCE-MRI parameters of bone metastases from HCC after RT and assessed their prognostic significance. Significant post-RT changes were noted in ADC, *k*_ep_, and *v*_*e*_. In addition, the percent change in ADC one month after RT was significantly different between the PD and non-PD groups, suggesting that it may help predict treatment response, which is considered to be unique to our study.

Several studies have suggested that pre- and posttreatment ADC could serve as a prognostic factor in various malignant tumors^20,37–39^, including HCC^40,41^. Our results were comparable to those of previous studies^20,25,37–41^, showing lower ΔADC% in the PD group. Furthermore, the ΔADC% could help differentiate between the PD and non-PD groups with 100% sensitivity and specificity using a cutoff of 6.79%, suggesting its potential as a predictor for early local tumor recurrence. Indeed, we acknowledge that validation of this cutoff value should be mandatory in future investigations, considering the repeatability of ADC measurements^18^ and intervendor differences^42^, which could be regarded as a limitation of DWI, and the small sample size of the present study; whether MRI can predict treatment response even earlier (e.g., within one month post-RT or during RT) or whether artificial intelligence and machine learning can predict treatment response are topics for future research. Regarding RT, there have been controversies regarding the optimal RT regimen for HCC bone metastasis^9,43^ and the dose–response relationship in HCC^44^. However, the high rates of up to 50% of retreatment following the use of conventional doses of RT^7^ have suggested the need for high-dose irradiation^9,10^. In this study, the crude rate of early local tumor progression 3 months after conventional RT was 40%. A subsequent boost with RT or early surgical interventions in patients showing a low ΔADC% at 1 month after the initial RT may improve local tumor control, and further studies are necessary to define optimal patient selection.

Similar to a previous study^45^, the baseline ADC of HCC bone metastases was relatively low in both the PD and non-PD groups, considering that the ADC of various pathologic bone marrow lesions generally ranged between 0.7 and 1.0 (× 10^–3^ mm^2^/s)^46–48^. With HCC being a hypervascular tumor, we considered that intratumoral hemorrhages within metastatic bone lesions may have contributed to the low ADC^12^. Unexpectedly, baseline ADC tended to be higher in the PD group, in contrast to previous studies that reported lower baseline ADC to be a risk factor for early recurrences or incomplete responses^49,50^. However, studies with contrasting results have also been reported, with higher baseline ADC values showing poor responses to chemotherapy or RT^38,51,52^. As necrotic tumors are less sensitive to chemotherapy or RT^52^, poor responses with higher baseline ADCs are likely to result from tumor necrosis. Although pseudo-diffusion could be another possible explanation^12^, its contribution is unlikely, as no significant differences were noted between DCE-MRI parameters of the two groups^53^.

It has been suggested that DCE-MRI parameters have potential as biomarkers for predicting prognoses and detecting treatment responses^54–57^. Regarding bone lesions, the *v*_*p*_ and *K*^trans^ decreased after RT, with *v*_*p*_ being the most strong predictor of treatment responses^27,58,59^. In contrast, we observed a significant decrease in *k*_ep_ and an increase in *v*_*e*_ after treatment; *K*^trans^ showed no significant change, possibly because *k*_ep_ and *v*_*e*_ changed in opposite directions. Furthermore, none of the DCE-MRI parameters could differentiate between the PD and non-PD groups, contrary to our expectation that they may also serve as prognostic factors for metastatic bone lesions from HCC. Although irrelevant to clinical outcomes, their significant changes implied that they can reflect pathophysiological changes after RT. As tumor cellularity and volume of extravascular extracellular space are inversely correlated^60,61^, it was reasonable that *v*_*e*_ decreased and ADC increased after RT. Meanwhile, the discrepancy observed between ADC and *v*_*e*_ in terms of their predictive values may be explained by the different extravascular extracellular space-related tumor environments^23,62^. In addition, we speculated that the method of ROI placement in our study could be one of the contributing factors for the negative results regarding DCE-MRI parameters, considering that previous studies placed ROIs mostly around hot spots representing a higher overall perfusion^58,59^. While the desirable placement of a ROI for tumor analysis remains debatable, tumor vascularity may have been underestimated in our study by the whole tumor assessment that did not exclude non-enhanced necrotic areas^63^. Nonetheless, we believe that our method using a multimodal tumor tracking application is one of the strengths of this study as it is less biased by the ROI choice and ensures the same ROI placement among different MRI sequences. Scanner, software, or operator-dependent variabilities, which are limitations in DCE-MRI^64^, or inhomogeneous responses between the intraosseous and extraosseous components^65^ can also be potential factors for the negative results that are contradictory to those of previous studies^27,58,59^.

Although there was no significant difference in volume when the whole study sample was assessed, some tumors showed an apparent increase in volume in the first post-RT MRI. Among the five patients who showed an increased post-RT tumor volume, only two were categorized into the PD group, which is partially comparable to the phenomenon termed “pseudo-progression”^66^. Pseudo-progression, first described in brain gliomas after RT and chemotherapy, is defined as treatment-related transient tumor growth^67^. Although there have been reports regarding pseudo-progression of bone lesions after high-dose stereotactic radiosurgery^66^, our results may imply that pseudo-progression can occur even after conventional dose regimens for bone metastasis from HCC. Further large-scale studies are necessary to validate these results.

Our study had several limitations. First, the sample size was limited to only ten patients, which may have influenced the reliability of the results, and the lack of multivariable analyses owing to the small sample size prohibited the determination of whether the predictive value of ΔADC% was independent of other MRI and clinical variables. Second, we used average DW- and DCE-MRI parameters calculated by two readers. However, owing to the high interobserver agreement, there was partial justification for the adoption of this method. Third, the physiology of individual patients may not have been appropriately reflected in the DCE-MRI parameters that were calculated based on a population-averaged AIF; although this method may have been advantageous in terms of reproducibility^32^. Fourth, the use of 0 s/mm^2^ as the first *b* value instead of 50 s/mm^2^ may have led to perfusion-related contributions to the ADC measurement^12^. Fifth, there may have been a mismatch of ROI between sequences. In particular, different slice thicknesses may have potentially resulted in discrepancies at the periphery of the lesions. Finally, the inclusion of both the enhanced and non-enhanced areas may have influenced the study results.

In conclusion, ADC and quantitative DCE-MRI parameters of metastatic bone lesions from HCC changed significantly in post-RT MRI. The percent change in ADC in early post-RT MRI can be used to evaluate treatment responses and may also predict local tumor progression. Future studies with larger patient populations and long-term clinical outcome evaluations are necessary to validate these findings.

## Abbreviations

ADC: Apparent diffusion coefficient
DCE: Dynamic contrast-enhanced
RT: Radiation therapy
HCC: Hepatocellular carcinoma
*K*^trans^: Volume transfer constant
*k*_ep_: Rate constant
*v*_*e*_: Volume fraction of the extravascular extracellular matrix
*v*_*p*_: Blood plasma volume
DW: Diffusion weighted
T1WI: T1-weighted image
T2WI: T2-weighted image
ROI: Regions of interest
CR: Complete response
PR: Partial response
PD: Progressive disease
SD: Stable disease
NRS: Numeric rating scale
OMED: Oral morphine equivalent dose
ROC: Receiver operating characteristic
AUC: Area under ROC curve
ICC: Interclass correlation coefficient
